# The intriguing plant nuclear lamina

**DOI:** 10.3389/fpls.2014.00166

**Published:** 2014-04-29

**Authors:** Malgorzata Ciska, Susana Moreno Díaz de la Espina

**Affiliations:** Department of Cell and Molecular Biology, Biological Research Centre – Consejo Superior de Investigaciones CientíficasMadrid, Spain

**Keywords:** plant nuclear envelope, plant nuclear lamina, LINC proteins, NMCP proteins, CRWN proteins, SUN proteins, Nup136, plant nucleocytoplasmic linkers

## Abstract

The nuclear lamina is a complex protein mesh attached to the inner nuclear membrane (INM), which is also associated with nuclear pore complexes. It provides mechanical support to the nucleus and nuclear envelope, and as well as facilitating the connection of the nucleoskeleton to the cytoskeleton, it is also involved in chromatin organization, gene regulation, and signaling. In metazoans, the nuclear lamina consists of a polymeric layer of lamins and other interacting proteins responsible for its association with the INM and chromatin. In plants, field emission scanning electron microscopy of nuclei, and thin section transmission electron microscopy of isolated nucleoskeletons, reveals the lamina to have a similar structure to that of metazoans. Moreover, although plants lack lamin genes and the genes encoding most lamin-binding proteins, the main functions of the lamina are fulfilled in plants. Hence, it would appear that the plant lamina is not based on lamins and that other proteins substitute for lamins in plant cells. The nuclear matrix constituent proteins are the best characterized structural proteins in the plant lamina. Although these proteins do not display strong sequence similarity to lamins, their predicted secondary structure and sub-nuclear distribution, as well as their influence on nuclear size and shape, and on heterochromatin organization, suggest they could be functional lamin analogs. In this review we shall summarize what is currently known about the organization and composition of the plant nuclear lamina and its interacting complexes, and we will discuss the activity of this structure in the plant cell and its nucleus.

The nuclear lamina is a ubiquitous structure that can be observed by transmission electron microscopy (TEM), forming a fibrous layer between the nuclear envelope (NE) and the condensed chromatin masses in many eukaryote cells, including those of protozoa and metazoans (**Figure 1**; Fawcett, 1966). The nuclear lamina is associated to the inner nuclear membrane (INM) and the inner side of the nuclear pore complexes (NPCs; Goldberg et al., 2008a, b; Gerace and Huber, 2012), and it is a prominent component of the nucleoskeleton (Simon and Wilson, 2011). The functions of the lamina are well established: it provides mechanical support for the nucleus and NE, it promotes the association between the nucleoskeleton and the cytoskeleton, facilitating nuclear movement and migration, and it is also involved in many activities that occur in the nucleus, such as chromatin organization and regulation and signaling (Gerace and Huber, 2012).

**FIGURE 1:**
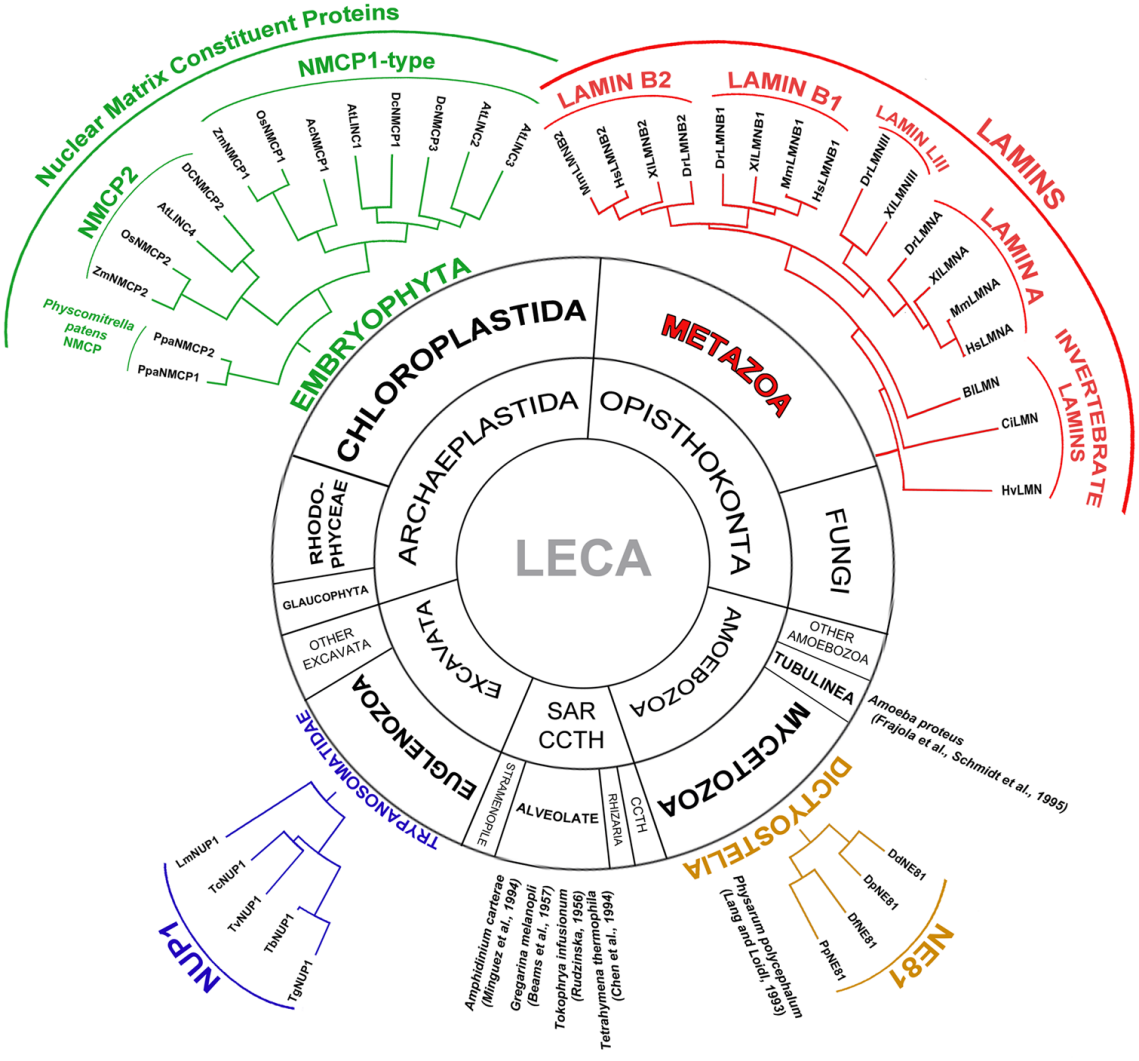
**Presence of the lamina in Eukaryota and classification and phylogenetic relationships between the lamina proteins in the different groups**. The lamina has been described in different Eukaryotic groups: Metazoan, Embryophyta, Dictyostelia, Trypanosomatidae, Alveolate and Tubulinea, although its constituent proteins differ in different groups. The main components of the metazoan lamina are lamins. Most invertebrates express a single lamin while vertebrates contain four genes encoding lamin B1, lamin B2, lamin A, and lamin LIII (lost in mammals; Peter and Stick, 2012). In Dictyostelia the lamina is made up of nuclear envelope 81 (NE81) protein, which is considered an ancestor of lamins (Batsios et al., 2012; Kruger et al., 2012). The lamina was also reported in the nucleus of various Alveolate species: *Amphidinium carterae*, *Gregarina melanopli*, *Tokophrya infusionum*, *Tetrahymena thermophila*, and in *Amoeba proteus* (Tubulinea) and *Physarum polycephalum* (Dictyostelia) that do not contain a gene encoding the NE81 protein. The composition of the lamina in these species is not known. In Trypanosomatidae it is made up of a single nuclear periphery 1 (NUP1) protein (Rout and Field, 2001; Dubois et al., 2012). In Embryophyta the lamina is made up of nuclear matrix constituent proteins (NMCPs; Masuda et al., 1997; Ciska et al., 2013). NMCPs are classified in flowering plants into NMCP1-type proteins and NMCP2. Monocots have one NMCP1 and one NMCP2 proteins while dicots contain one NMCP2 and two or three NMCP1-type proteins. The moss *Physcomitrella patens* contains two NMCP proteins (Ciska et al., 2013). Selected species for the representation of the NMCP protein family: *Allium cepa* (Ac), *Arabidopsis thaliana* (At), *Daucus carota* (Dc), *Oryza sativa* (Os), *Physcomitrella patens* (Ppa), and *Zea mays* (Zm); NE81 proteins: *Dictyostelium discoideum* (Dd), *Dictyostelium fasciculatum* (Df), *Dictyostelium purpureum* (Dp), and *Polysphondylium pallidum* (Pp); NUP1 proteins: *Trypanosoma brucei* (Tb), *Trypanosoma gambiense* (Tg), *Trypanosoma cruzi* (Tc), *Trypanosoma vivax* (Tv), and *Leishmania major* (Lm); and lamins: *Hydra vulgaris* (Hv), *Ciona intestinalis* (Ci), *Branchiostoma lanceolatum* (Bl), *Danio rerio* (Dr), *Xenopus laevis* (Xl), *Mus musculus* (Mm), and *Homo sapiens* (Hs). LECA, the last eukaryotic common ancestor; SAR, stramenopile, alveolate, Rhizaria; CCTH, cryptomonads, centrohelids, telonemids, haptophytes.

The metazoan lamina is a complex protein mesh that consists of a polymeric layer of lamins, intermediate filament proteins that associate with numerous transmembrane lamin-binding proteins that anchor the lamina to the INM, as well as chromatin associated factors that tether chromatin to this structure (Ho and Lammerding, 2012; Simon and Wilson, 2013). Plants contain a nuclear lamina with a similar organization to that of metazoans (Fiserova et al., 2009; Moreno Diaz de la Espina, 2009), even though plant genomes lack genes that code for lamins and lamin-binding proteins, except for the Sad1/UNC84 (SUN) domain proteins (Mans et al., 2004; Rose et al., 2004; Graumann et al., 2013) that participate in LINC (linker of the nucleoskeleton to cytoskeleton) complexes which bind the nucleoskeleton to the cytoskeleton in metazoan. In light of the crucial roles played by the lamina and by lamins in the nucleus and the cell, and given that the plant lamina is not lamin-based, many studies have focused on this structure and on the characterization of its ultrastructural and protein composition (Masuda et al., 1993, 1997; Dittmer et al., 2007; Fiserova et al., 2009; Ciska and Moreno Díaz de la Espina, 2013; Ciska et al., 2013; Sakamoto and Takagi, 2013). In this review, we use the term lamina to refer to the complex filamentous protein network associated with the INM, chromatin, nucleocytoplasmic bridging complexes, and the NPCs following the conventions applied for other eukaryotes including those that lack lamin genes. We also establish what is currently known about the structure and nature of the plant lamina, and we consider its implication in some of the activities undertaken by the metazoan lamina, such as the regulation of nuclear size and shape and chromatin organization, and also the physical connections established between the nucleoskeleton and cytoskeleton.

## THE METAZOAN LAMINA

Although the first descriptions of the lamina in protozoa date from the 1950s (Pappas, 1956; Beams et al., 1957), it was not until it was described in mammalian cells that interest in the lamina became more widespread (Fawcett, 1966). Thin section TEM shows the lamina to be a thin fibrillar layer between the NE and the condensed chromatin masses (Pappas, 1956; Beams et al., 1957; Fawcett, 1966). The fibrous nature of the lamina was corroborated when its fibrils were seen to interconnect with the NPCs in NE fractions from amphibian oocytes (Scheer et al., 1976) and rat liver cells (Aaronson and Blobel, 1975; Dwyer and Blobel, 1976). A decade later, the well organized filament network of detergent extracted NEs from *Xenopus laevis* oocytes was shown by TEM to have a crossover spacing of about 50 nm after metal shadowing (Aebi et al., 1986). Since then, the ultrastructural organization of the metazoan lamina has been characterized in amphibian oocyte NEs by feSEM (field emission scanning electron microscopy) as a regular orthogonal network of 10 nm filaments connected to the NPCs (Goldberg et al., 2008a, b). However, studying the filamentous network that constitutes the lamina in somatic cells is difficult due to its association with chromatin, the lamina displaying a more irregular structure in these preparations (Goldberg et al., 2008b).

The lamina was first isolated in the 1970s from rat liver nuclei, in which a conspicuous lamina could not be observed by thin section conventional TEM (Aaronson and Blobel, 1975). Its three main polypeptides were identified (Gerace et al., 1978) and later called lamins (Gerace and Blobel, 1980). Lamins are ancestral members of the highly conserved intermediate filament protein superfamily (Mckeon et al., 1986; Franke, 1987; Peter and Stick, 2012) and they have the same typical tripartite structure, with a central coiled coil domain formed by four coils that are separated by short linkers. Flanking the coiled coil domain, lamins have a short N-terminus that contains a conserved phosphorylation site for cdk1 and a longer globular C-terminal tail with a second conserved cdk1 site, as well as a nuclear localization signal (NLS), a highly conserved Ig fold and a C-terminal cysteine, aliphatic residues, any amino acid (CAAX) box (Dechat et al., 2008, 2010; Dittmer and Misteli, 2011).

All metazoans express lamins and while invertebrates contain one or two lamin genes, there are three or four in vertebrates. Lamins have been classified as types A or B according to their structure, distribution, mitotic behavior, and biochemical characteristics (Dittmer and Misteli, 2011; Ho and Lammerding, 2012). Most invertebrates have a single type B lamin gene (Melcer et al., 2007; Peter and Stick, 2012), while the three vertebrates type B lamin genes are complemented with a forth lamin A gene that encodes lamin A and an alternative splicing product, lamin C found in mammals that have lost the gene for lamin LIII (**Figure 1**; Peter and Stick, 2012). Except for lamin C, all lamins are expressed as prelamins and they undergo highly regulated and extensive post-translational modification of the CAAX box through farnesylation, proteolytic cleavage, and carboxylation. Type B lamins remain permanently modified while the 15 terminal amino acids of prelamin A are removed to produce the mature lamin A that lacks the modification (Dechat et al., 2010; Simon and Wilson, 2013). Lamins also undergo other post-translational modifications such as sumoylation and phosphorylation. Conserved phosphorylation sites for different kinases are involved in the polymerization and mitotic disassembly of lamins, as well as in the regulation of conserved functions. By contrast, unique phosphorylation sites probably mediate the differential regulation of lamins in different tissues (Simon and Wilson, 2013).

Besides lamins, the lamina contains numerous associated proteins, most of which are transmembrane proteins of the INM that bind to lamins and promote the association of the lamina with the NE. In addition, lamin-binding proteins may interact with DNA and some chromatin proteins, organizing the positioning of chromatin at the NE. Thus, lamins interact with numerous structural and regulatory proteins, many of which have mechanical and structural roles: stabilizing the lamina and anchoring lamin filaments to the INM; linking the lamina to the cytoskeleton; anchoring the lamina to NPCs; and tethering chromatin to the INM. In addition, some of these proteins regulate signaling and transcription. The lamin-binding proteins have been studied extensively (Wilson and Foisner, 2010; Ho and Lammerding, 2012; Simon and Wilson, 2013) and to date, in humans 54 binding partners have been identified for lamin A, 23 for lamin B1, and seven for lamin B2. Indeed, the functional association of many of these partners have been confirmed using molecular biology tools, including that of LEM (lamin, emerin, MAN) domain proteins, BAF (barrier to autointegration factor), Rb (retinoblastoma), and SUN domain proteins (Simon and Wilson, 2013). The partners of lamin A are involved in different nuclear activities and they include components of the nucleoskeleton and NPCs, such as lamins B1 and B2, actin, nesprin1α and nesprin2, SUN1 and SUN2, nucleoporins Nup153 and Nup88, LCO1 (lamin companion 1). In addition, lamin A can associate with LEM domain proteins like LAP2a, MAN1, LEM2 and emerin, which are integral INM proteins that interact with lamins and BAF, and that form complexes involved in nuclear architecture and in anchoring chromatin to the NE. Other partners include chromatin associated proteins, such as BAF, PCNA, HP1 and histones, as well as transcription factors like Rb or other proteins involved in transcription and signaling (Simon and Wilson, 2013). The SUN proteins associate with Klarsicht/ANC1/syne homology (KASH) domain proteins of the outer nuclear membrane (ONM), forming the core of the LINC complex that associates with the cytoskeleton (Sosa et al., 2012; Tapley and Starr, 2013). In this way, the lamin polymer would constitute a base for the supramolecular assembly that connects the cytoskeleton with the NE and chromatin.

Although the process of lamin self assembly has been described relatively well *in vitro* for chicken, human and *Caenorhabditis elegans* lamins, the supramolecular assembly of the higher order arrays of lamins with their multiple associated proteins remains somewhat unclear due to the difficulty of reconstituting the NE environment *in vitro*. Lamin polymerization involves lamin dimerization, the longitudinal assembly of these dimers into oligomers that can interact laterally to form protofilaments, and the further assembly of these as 10 nm filaments (Ben-Harush et al., 2009; Dittmer and Misteli, 2011). The rod domains play important roles in lamin homodimerization and in the formation of lateral and longitudinal contacts (Kapinos et al., 2010; Gangemi and Degano, 2013). However, there is little information regarding how lamins are incorporated into the lamina *in vivo* and most of this comes from studies on the amphibian oocyte lamina. *In vivo*, lamins form an orthogonal mesh in the lamina connected to the inner ring of NPCs (Goldberg et al., 2008a, b; Gerace and Huber, 2012), whereby type A and B lamins form separate filament networks, although these may interact to varying degrees (Goldberg et al., 2008a, b; Kolb et al., 2011). FRAP (fluorescence recovery after photobleaching) analysis demonstrates that lamins are stably integrated into the lamina and along with lamin-associated transmembrane proteins, although the latter are more mobile than lamins (Moir et al., 2000; Ostlund et al., 2006).

The lamina is involved in many nuclear and cellular functions that are fulfilled by its multiple lamin-dependent complexes. The lamina fulfills several structural functions, regulating the size, shape, and mechanical properties of the nucleus, stabilizing the NE, positioning the NPC, mediating the physical connection between the nucleus and cytoskeleton, and positioning heterochromatin at the NE. However, it is also involved in other processes, including epigenetic modification, chromatin organization, DNA replication, repair and transcription, as well as cell proliferation, and differentiation (Dechat et al., 2008, 2009, 2010; Ho and Lammerding, 2012; Burke and Stewart, 2013).

## THE LAMINA IN NON-METAZOANS

As mentioned above, the nuclear lamina is not a structure that is only found in metazoans that express lamins. A well organized lamina has been identified by TEM in several Protozoa species from diverse groups, including phylogenetically unrelated unicellular eukaryotes (Frajola et al., 1956; Pappas, 1956; Rudzinska, 1956; Beams et al., 1957; Lang and Loidl, 1993; Chen et al., 1994; Minguez et al., 1994), and it has also been isolated from *Trypanosoma* (Rout and Field, 2001; **Figure 1**). Except for that in *Amoeba proteus* and *Gregarina melanopli* (Frajola et al., 1956; Beams et al., 1957; Schmidt et al., 1995), the protozoan lamina resembles that of metazoans, particularly once isolated (Rout and Field, 2001). However, protozoa lack lamin orthologs and thus, their lamina is likely to be based on different proteins with similar functions. Yet to date, only two constituent proteins of the protozoan lamina that fulfill similar functions to lamin have been characterized in *Dictyostelium discoideum* and *Trypanosoma brucei* (Batsios et al., 2012; Dubois et al., 2012; Kruger et al., 2012).

Dictyostelids belong to a group of Amoebozoa that are relatively close to metazoans. The *Dictyostelium* lamin-like protein NE81 is restricted to the class Dictyostelia (**Figure 1**) and it is currently considered to be an evolutionary precursor of metazoan lamins, in particular given that it shares important structural and functional features with them, such as: size; the distribution of the coiled coils in the rod domain; the position of the cdk1 phosphorylation consensus site preceding the rod domain; the NLS in the tail; and the terminal CAAX box. Moreover, the generation of knockout and over-expression mutants has demonstrated that like lamins, NE81 plays an important role in maintaining nuclear integrity, chromatin organization, and the mechanical stability of cells (Batsios et al., 2012; Kruger et al., 2012).

Trypanosomatids are highly divergent unicellular eukaryotes and in *T. brucei*, NUP-1 has been shown to be the major component of the isolated lamina (Rout and Field, 2001). NUP-1 is restricted to trypanosomatids, which have a single NUP-1 ortholog (**Figure 1**). This is a 400 kDa long coiled coil protein containing 20 repeats of a 144 amino acid sequence. NUP-1 is not related to lamins but it does share structural features with them, and it is also implicated in processes controlled by lamins, such as: the regulation of nuclear shape and size, the distribution of NPCs, heterochromatin organization and epigenetic control of developmentally regulated genes (Rout and Field, 2001; Dubois et al., 2012). Accordingly, the trypanosomatid lamina appears to be based on NUP-1, even though this is a protein phylogenetically unrelated to lamins.

In conjunction, the above indicates that the lamina is a ubiquitous nuclear structure with conserved functions in eukaryotes, yet the proteins that constitute the lamina in Protozoa, a group that includes phylogenetically unrelated unicellular eukaryotes, might have evolved separately from those that make up this structure in metazoans.

## THE PLANT LAMINA

**FIGURE 2:**
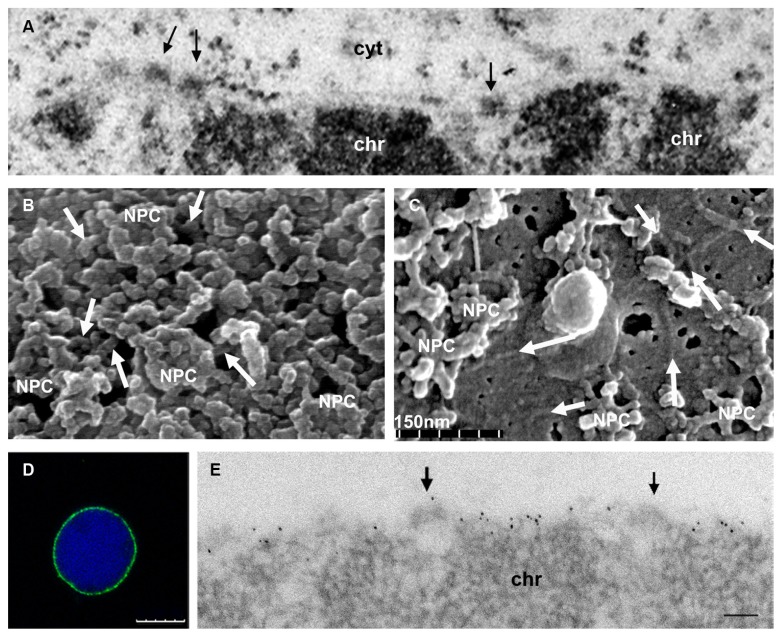
**Ultrastructure of the plant nuclear lamina and localization of the NMCP proteins. (A)** Conventional thin section TEM image of the nuclear periphery of an onion meristematic root cell, showing a portion of the NE with its two membranes, and the dense NPCs (arrows) that traverse it. The heterochromatin (chr) is tightly attached to the INM but the thin lamina is not conspicuous with this technique. Cytoplasm (cyt). **(B)** Cytoplasmic face of the NE of a tobacco BY-2 cell nucleus extracted with Triton X-100 to remove the membranes and visualized by feSEM. The filaments of the lamina interconnecting the NPCs are evident (arrows). **(C)** feSEM image of the nucleoplasmic face of the NE of a BY-2 nucleus that has been fractured but not extracted with Triton X-100. Arrows indicate the filaments of the lamina in the membrane. **(D,E)** Detection of NMCP1 in the lamina of isolated meristematic onion nuclei extracted with Triton X-100 after immunofluorescence and DAPI staining **(D)**, or TEM immunogold labeling **(E)**. After removing the membrane, the lamina with a lower electron density than chromatin and containing NMCP1 proteins is evident at the nuclear periphery. The association with NPCs (arrows) and the tight attachment to the condensed chromatin masses (chr) can be seen. (**B,C** courtesy of Drs J. Fiserova and M. W. Goldberg). Bars in *D* = 10 μm and in *E* = 100 nm.

Although conventional thin section TEM of plant cells does not reveal a conspicuous lamina underlying the nucleoplasmic side of the NE (**Figure 2A**), a peripheral fibrillar layer with associated NPCs was evident in demembrated nuclei (**Figure 2E**), similar to the metazoan lamina, as well as in the nucleoskeleton of both monocot and dicot cells after the elimination of membranes, chromatin and soluble proteins from the nucleus (Moreno Diaz de la Espina et al., 1991; Li and Roux, 1992; Masuda et al., 1993; Minguez and Moreno Diaz de la Espina, 1993; Moreno Diaz de la Espina, 1995, 2009). Moreover, an analysis of the plant NE by feSEM confirmed the presence of a lamina similar to that of metazoans attached to the INM that was called plamina (Fiserova et al., 2009; Fiserova and Goldberg, 2010). With this technique the plant lamina appears to be a complex, organized filamentous structure that underlies the INM and that is connected to the nucleoplasmic ring of the NPCs (**Figures 2B,C**). Well defined tightly packed filaments were observed at the INM of tobacco cells, suggesting that the lamina would be formed by proteins that can form filaments (**Figure 2C**). The filaments in the lamina were 10–13 or 5–8 nm thick, similar dimensions to those observed in isolated lamina fractions from pea nuclei (Li and Roux, 1992; Blumenthal et al., 2004; Fiserova et al., 2009). On view of the structural similarities of the metazoan and plant lamina, the specific term plamina recently coined for the plant lamina is not necessary, adds confusion to the field and should be avoided.

## PROTEIN COMPONENTS OF THE PLANT LAMINA

Despite the structural similarities of the plant and metazoan lamina, plants lack orthologs of lamins and of the lamin-binding proteins, except for the SUN domain proteins that are conserved in all kingdoms (Mans et al., 2004; Rose et al., 2004; Moriguchi et al., 2005; Graumann et al., 2010; Murphy et al., 2010; Field et al., 2012). However, the critical functions performed by lamins and their partners in metazoan cells (see above) are fulfilled in the plant cell. Hence, the plant lamina must be established by proteins that evolved separately to those in metazoans, as is the case of the NUP-1-based lamina in trypanosomids (**Figure 1**; Rout and Field, 2001; Dubois et al., 2012). Similarly, such proteins would represent functional plant homologs of lamins, with similar characteristics rather than sequences. These lamin-like proteins should have a coiled coil structure similar to lamins and NUP-1, and they should be able to form filaments, to become stably integrated into the nucleoskeleton, and to participate in structural and biochemical interactions related to the formation of networks and multiprotein complexes.

Since the discovery of the plant lamina several insoluble proteins have been proposed as putative plant lamin-like proteins, mainly based on their localization in the lamina, their cross reactivity with vertebrate lamins and intermediate filaments (Li and Roux, 1992; Minguez and Moreno Diaz de la Espina, 1993; Moreno Diaz de la Espina, 1995, 2009), and on their ability to form filaments *in vitro* (Blumenthal et al., 2004). However, the sequences of these proteins are still not available to analyze and compare them with lamins.

The best candidates to fulfill the functions of lamins in plants are the NMCPs (nuclear matrix constituent proteins) that in *Arabidopsis thaliana* were later called LINC (little nuclei) and very recently renamed as crowded nuclei (CRWN; **Table 1**), known to be components of the lamina (**Figures 2D,E**; Masuda et al., 1993; Ciska et al., 2013; Ciska and Moreno Díaz de la Espina, 2013; Sakamoto and Takagi, 2013). These proteins have a tripartite structure with a central coiled coil domain, and they are predicted to dimerize and probably form filaments like lamins (Masuda et al., 1999; Dittmer et al., 2007; Ciska et al., 2013; Ciska and Moreno Díaz de la Espina, 2013). They also participate in the nuclear functions mediated by lamins, such as the regulation of nuclear shape and size, and heterochromatin organization (Dittmer et al., 2007; Dittmer and Richards, 2008; van Zanten et al., 2011, 2012; Sakamoto and Takagi, 2013; Wang et al., 2013). NMCPs are highly conserved in land plants (Ciska et al., 2013; Ciska and Moreno Díaz de la Espina, 2013) and although they do not share sequence similarity with lamins, their predicted structure and subnuclear distribution suggest that they participate in the formation of the plant lamina network.

**Table 1:** Terminology used for NMCP proteins reported in different plant species.

Species	Accession number	Protein	Reference
*Daucus carota* (carrot)	BAA20407	DcNMCP1	Masuda et al. (1993, 1997), Ciska et al. (2013), Ciska and Moreno Díaz de la Espina (2013), Kimura et al. (2014)
	BAI67718	DcNMCP2	Kimura et al. (2010), Ciska et al. (2013), Ciska and Moreno Díaz de la Espina (2013)
	BAN14787	DcNMCP3	Ciska and Moreno Díaz de la Espina (2013)
*Apium graveolens* (celery)	BAI67715	AgNMCP1	Kimura et al. (2010), Ciska et al. (2013), Ciska and Moreno Díaz de la Espina (2013)
	BAI67716	AgNMCP2	Kimura et al. (2010), Ciska et al. (2013), Ciska and Moreno Díaz de la Espina (2013)
*Oryza sativa* (rice)	AB110204	OsNMCP1	Moriguchi et al. (2005), Ciska et al. (2013), Ciska and Moreno Díaz de la Espina (2013)
	AB110205	OsNMCP2	Ciska et al. (2013), Ciska and Moreno Díaz de la Espina (2013)
*Allium cepa* (onion)	AB673103	AcNMCP1	Ciska et al. (2013), Ciska and Moreno Díaz de la Espina (2013)
*Arabidopsis thaliana*	At1g67230	NMCP1 like	Rose et al. (2004)
		LINC1	Dittmer et al. (2007), Ciska et al. (2013), Sakamoto and Takagi (2013)
		CRWN1	Wang et al. (2013)
	At1g13220	NMCP1 like	Rose et al. (2004)
		LINC2	Dittmer et al. (2007), Ciska et al. (2013), Sakamoto and Takagi (2013)
		CRWN2	Wang et al. (2013)
	At1g68790	NMCP1 like	Rose et al. (2004)
		LINC3	Dittmer et al. (2007), Ciska et al. (2013), Sakamoto and Takagi (2013)
		CRWN3	Wang et al. (2013)
	At5g65770	NMCP1 like	Rose et al. (2004)
		LINC4	Dittmer et al. (2007), Ciska et al. (2013), Sakamoto and Takagi (2013)
		CRWN4	Wang et al. (2013)

## NMCP PROTEINS, THE PLANT ANALOGS OF LAMINS

The first NMCP protein (DcNMCP1) was described as a residual 130 kDa protein component of the carrot nuclear matrix (Masuda et al., 1993). The determination of its cDNA sequence enabled its structure to be predicted, similar to that of lamins with a central coiled coil domain predicted to mediate dimerization and a NLS in the tail domain (Masuda et al., 1997). Also DcNMCP1 assembled and disassembled in mitosis as occurs with lamins (Masuda et al., 1999). NMCP1 was later isolated and characterized in a monocot, *Oryza sativa* (Moriguchi et al., 2005). Another homolog, NMCP2, was later identified in carrot and celery (Kimura et al., 2010), and four homologs were identified in a genome-wide search for coiled coil proteins in *A. thaliana* (Rose et al., 2004), which were later called LINC (little nuclei) 1–4 due to the phenotype of the corresponding mutants (Dittmer et al., 2007). However, this name was misleading as it had already been attributed to the LINC complex of the NE (Crisp et al., 2006). On view of this, the same group recently renamed the proteins as CRWN according to another phenotype of the mutants (Wang et al., 2013) which adds more confusion to the nomenclature of the proteins. In our opinion the original terminology of NMCP is more appropriate not only because it was the first adopted and is currently in use for all species but *A. thaliana* (**Table 1**), but also because it refers to an intrinsic feature of the proteins. For the purposes of this review we will use the original terminology of NMCP and LINC/CRWN only for *A. thaliana* proteins, but the importance of the proteins and the expected future development of the field deserve an agreement of the different groups involved to establish a common terminology.

Recent searches of plant genomes have revealed that *NMCP* genes are conserved in land plants, which contain genes coding for two or more NMCP proteins (Kimura et al., 2010; Ciska et al., 2013; Ciska and Moreno Díaz de la Espina, 2013). NMCPs constitute a highly conserved family of proteins in plants, except in single cell plants, and they are absent from metazoans and fungi (Ciska et al., 2013; Ciska and Moreno Díaz de la Espina, 2013). NMCP proteins have been classified into two clusters following their first denominations: NMCP1 and NMCP2 (**Figure 1**; Masuda et al., 1993, 1997; Kimura et al., 2010; Ciska et al., 2013; Ciska and Moreno Díaz de la Espina, 2013; Wang et al., 2013). Monocots have one *NMCP1* and one *NMCP2* gene, while dicots carry a single *NMCP2* gene and several *NMCP1*-type genes encoding NMCP1-related proteins called NMCP1 and NMCP3. *A. thaliana* and some other dicots contain two *NMCP3*-type genes, while two *Solanum* species, *Solanum tuberosum* and *S. lycopersicum* have two *NMCP1* genes and no genes encoding *NMCP3*. *AtLINC1/CRWN1* encodes an NMCP1, whereas *AtLINC2/CRWN2* and *AtLINC3/CRWN3* encode NMCP3-type and *AtLINC4/CRWN4* encodes an NMCP2 protein (Ciska et al., 2013; Ciska and Moreno Díaz de la Espina, 2013; Wang et al., 2013). The two *NMCP* genes of the moss *Physcomitrella patens* evolved from a common *NMCP* progenitor gene and they are included in the *NMCP2* cluster, suggesting that the archetypal NMCP progenitor was actually an NMCP2 protein (Ciska et al., 2013; Ciska and Moreno Díaz de la Espina, 2013). All *LINC/CRWN* genes are expressed in whole *A. thaliana* plants (Sakamoto and Takagi, 2013) and their expression is developmentally regulated (Ciska et al., 2013; Ciska and Moreno Díaz de la Espina, 2013), as occurs with lamins (Benavente et al., 1985; Broers et al., 1997; Peter and Stick, 2012).

**FIGURE 3:**
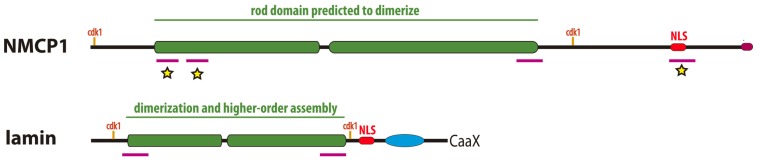
**Comparison of the structure of plant NMCP proteins and metazoan lamins**. Both proteins have a similar tripartite structure with a central coiled–coil domain (green boxes) flanked by a short head and a long tail domains. The rod domain which is responsible for dimerization and higher order assembly in lamins, presents highly conserved regions at both ends (magenta bars) involved in head to tail association of dimers in the case of lamins. The rod domain is flanked by conserved cdk1 phosphorylation sites in both cases. In the tail domain both have a NLS (red boxes) and a conserved C-terminus (magenta box in NMCP1 and CAAX box in lamins). NMCPs lack the Ig fold for partner protein binding typical of lamins (blue oval). The conserved regions marked with a yellow star are involved in NMCP1 association to the nuclear periphery (Kimura et al., 2014).

The predicted structure of NMCP proteins is well characterized (**Figure 3**; Ciska et al., 2013; Ciska and Moreno Díaz de la Espina, 2013). As indicated above, they have a tripartite structure similar to that of lamins with a highly conserved central coiled coil rod domain that is predicted to dimerize, and less conserved non-coiled coil head and tail domains. The structure and length of the rod domain are conserved across the NMCP family, suggesting that it plays an important role in oligomerization. Moreover, at each end of the rod domain of NMCPs and in the predicted linkers there are five highly conserved and family specific regions. Lamins have a similar distribution of conserved motifs although there is no significant sequence similarity between these two protein families (**Figure 3**). NMCPs also contain several conserved motifs in the tail domain, including a stretch of acidic amino acids that is also present in vertebrate lamins, a NLS and a NLS-linked conserved motif in NMCP1’s, both necessary for association of the protein to the NE (Kimura et al., 2014). The later is identical to a specific actin binding site in lamin A. NMCPs lack a CAAX box but their C-terminus is highly conserved, except for that in the dicot NMCP2 (Ciska et al., 2013). They also lack the Ig fold in the tail that is involved in the interaction of lamins with some of their protein partners (Simon and Wilson, 2013).

The general organization of the rod domain in NMCPs and lamins is similar, although the former is twice as long as that in lamins, with a similar distribution of conserved motifs, including those at their ends (Ciska and Moreno Díaz de la Espina, 2013) that mediate head to tail associations in lamins (Kapinos et al., 2010; Davidson and Lammerding, 2013). The analogous structures, the similar location of the conserved motifs in the rod domain and the presence of consensus sequences for cdk1 at either side of the rod domain, suggest a similar mechanism of oligomerization and protofilament formation for NMCPs and lamins (**Figure 3**). As yet, NMCP proteins have not been polymerized *in vitro* and their mechanisms of assembly in the lamina are poorly known. Very recently it has been established that DcNMCP1 associates to the nuclear periphery by coordinate action of the NLS and a NLS-linked conserved motif and the 141 amino acids at the N-terminus of the protein comprising the head and the highly conserved N-terminus of the rod domain (Kimura et al., 2014; see **Figure 3**). The N-terminal region of NMCP1 could be involved in head to tail assembly of dimers and polymer assembly stabilization as occurs in lamins (Davidson and Lammerding, 2013) and may be critical for the integration of the proteins in the lamina network. The immuno-feSEM experiments that are in progress should help to detect the NMCP1 protein in the filaments of the plant lamina.

The functions of NMCP proteins are poorly understood as the phenotypic effects of their mutations are not as severe as those caused by mutations in lamins (Butin-Israeli et al., 2012; Ho and Lammerding, 2012). NMCP proteins are involved in essential processes as quadruple NMCP/LINC/CRWN mutants are not viable. By contrast, single, double and some triple mutants are viable (Dittmer et al., 2007; Sakamoto and Takagi, 2013; Wang et al., 2013), which in conjunction with the lack of phenotype of single mutants, indicates that there is complementation between different proteins.

To date, the best analyzed function of NMCP/LINC/CRWN proteins is the regulation of nuclear size and shape (Dittmer et al., 2007; van Zanten et al., 2011; Sakamoto and Takagi, 2013; Wang et al., 2013), a function also fulfilled by lamins (Ho and Lammerding, 2012). NMCP/LINC/CRWN mutations result in a decrease in nuclear size and alterations to the shape of differentiated cells, with a predominant influence of the *LINC1/CRWN1* and *LINC4/CRWN4* genes (Dittmer et al., 2007; Dittmer and Richards, 2008; van Zanten et al., 2011; Sakamoto and Takagi, 2013; Wang et al., 2013). In addition, over-expression of LINC4 results in an increase in nuclear size (Sakamoto and Takagi, 2013), although the underlying molecular mechanisms involved remain unknown. Other proteins of the plant NE that also affect nuclear size and shape are those forming the nucleocytoplasmic linker in plants, such as the SUN domain proteins (Oda and Fukuda, 2011; Zhou et al., 2012), the KASH-like WIP (WPP domain interacting proteins) proteins (Zhou et al., 2012) and the WIT (WPP domain-interacting tail-anchored) proteins (Tamura et al., 2013), as well as nucleoporin Nup136 (Tamura et al., 2010; Tamura and Hara-Nishimura, 2011). These results suggest that the proteins forming the plant nucleocytoplasmic linker interact with NMCPs, as well as Nup136, a component of the nucleoplasmic basket of NPCs that has been proposed to link the NPC to the lamina in plants (Tamura and Hara-Nishimura, 2013), thereby cooperating in the regulation of nuclear morphology.

The role of NMCP proteins in chromatin organization remains unclear. A decrease in the number of chromocentres was reported in *linc1/crwn1-linc2/crwn2* mutants (Dittmer et al., 2007), although the relative heterochromatin fraction and the distribution of specific heterochromatin regions during seed germination was unaltered in these mutants (van Zanten et al., 2011, 2012). Nevertheless very recent results using different mutants showed that chromocentre organization is disrupted in *linc4/crwn4* mutants, as demonstrated by the dispersion of 5S RNA genes and centromeric repeat arrays (Wang et al., 2013). Accordingly, it was suggested that NMCP/LINC/CRWN proteins play a role in maintaining proper heterochromatin organization. Thus, NMCP1/LINC1/CRWN1 and NMCP3/LINC2/CRWN2 could prevent chromocentre aggregation while NMCP2/LINC4/CRWN4 would have a complementary role in maintaining chromocentre integrity (Wang et al., 2013).

Lamins mediate nuclear positioning and movement through an interaction between lamin A with SUN proteins, which associate with KASH proteins to form the metazoan LINC complex that connects the lamina to the cytoskeleton (Sosa et al., 2012; Tapley and Starr, 2013). Very recently, a nucleocytoplasmic linker has been described in plants, which is involved in nuclear positioning and movement in response to environmental stimuli. It consists of a plant-specific Myosin Xl-i motor that binds to the actin filaments of the cytoskeleton, and also to the WIT proteins in the ONM that form a complex with WIP proteins, in turn interacting with SUN proteins (Tamura et al., 2013). Thus, the complex formed by the WIT, WIP, and SUN proteins in plants would be analogous to the metazoan LINC complex. While connections between this complex and NMCP proteins (or other intranuclear components) are yet to be defined, an interaction between NMCP and SUN has been recently demonstrated (Graumann, 2014). The analysis of *linc/crwn1–4* and *linc/crwn2–3* mutants apparently rules out a role for NMCP proteins in blue light-induced nuclear movement (Sakamoto and Takagi, 2013), although protein complementation cannot be completely discarded.

As indicated above, NMCPs show many analogies to lamins (Ciska and Moreno Díaz de la Espina, 2013) and in the future, even more may emerge as our understanding of these proteins improves. For all these reasons, and also because they are conserved in all land plants and they localize in the lamina (Masuda et al., 1993, 1997; Dittmer et al., 2007; Ciska et al., 2013; Sakamoto and Takagi, 2013), NMCPs are considered to be the plant analogs of lamins and the main components of the filament mesh that constitutes the plant lamina.

## OTHER COMPONENTS OF THE PLANT LAMINA

**FIGURE 4:**
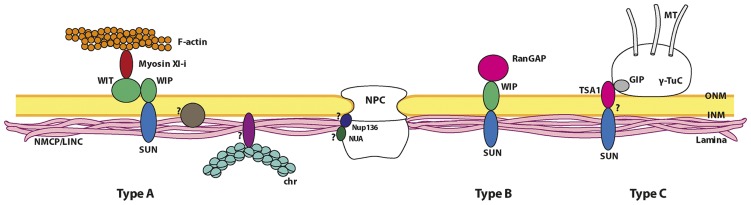
**Proposed model of the nuclear lamina organization and its main interacting partners in plants**. The plant lamina is made up of NMCP proteins and it is attached to the INM of the NE through its interaction with INM proteins not yet identified. The lamina is also attached to NPCs, probably through its interaction with Nup136 and NUA. The factors involved in chromatin association to the lamina in plants remain unknown. The lamina associates to plant-specific nucleocytoplasmic linkers probably by interaction of NMCPs with SUN proteins, which are currently divided into three different types. The type A linker can be considered as a plant LINC complex because it connects the lamina with the actin cytoskeleton. The core organization of this complex is similar to that of type B linkers. The linker element that interacts with the cytoskeleton is myosin Xl-i, which binds to both the perinuclear actin filaments and the WIT protein in the ONM, that in turn interacts with the SUN–WIP bridge (Tamura et al., 2013). The type B linker is formed by SUN proteins anchored to the INM that form a bridge with the WIPs in the ONM, which may in turn complex with WIT proteins in some cases. This type of complex is necessary for RanGAP to associate with the NE (Zhou et al., 2012; Zhou and Meier, 2013). Recently a model for the attachment of γ-TuCs (γ-tubulin complexes) to the NE has been proposed (type C linker). In this complex, the interaction of a small component, GIP (GCP3-interacting protein), with TSA1, an ONM protein that contains a VIPt motif similar to the φ-VPT motif of WIPs, would facilitate an interaction with SUNs (Batzenschlager et al., 2013). Proteins in the different complexes are represented as monomers for simplification. Non-proven interactions are indicated by question marks.

While the plant lamina and its main structural components, the NMCP proteins, have now been relatively well characterized, the proteins anchoring this structure to the INM, NPCs, chromatin and the cytoskeleton remain largely unknown. Plants lack orthologs of the metazoan lamin-interacting proteins that attach the lamina to the INM, such as the LBR (lamin B receptor), LEM domain proteins and nesprins, or to NPCs, such as Nup153 (Mans et al., 2004). Hence, it would appear likely that they have evolved specific NMCP-interacting proteins that anchor the lamina to the NE and NPCs, also participating in the attachment of chromatin, and in the control of other nuclear and cellular activities regulated by lamins in metazoans. Searching for the partners of NMCPs in the lamina is fundamental to understand the functions and organization of this structure, yet to date, only one NMCP binding protein has been unequivocally identified (Graumann, 2014), even though the functional analysis of mutants suggests that direct or indirect interactions could occur with several proteins in the NPCs and NE, such as Nup136 (Tamura and Hara-Nishimura, 2011), SUN proteins (Graumann et al., 2010; Oda and Fukuda, 2011; Zhou et al., 2012; Graumann, 2014); WIPs (Zhou et al., 2012) and WITs (Tamura et al., 2013; **Figure 4**).

## CONNECTION OF THE PLANT LAMINA WITH THE NPCs

As occurs in metazoa (Aaronson and Blobel, 1975) and protozoa (Rout and Field, 2001), NPCs associate with the isolated plant lamina (Moreno Diaz de la Espina, 2009). Moreover, feSEM analysis of the plant NE suggests that a direct interaction may occur between the filaments of the plant lamina and the inner ring of the NPCs (**Figure 2C**; Fiserova et al., 2009). However, the proteins that mediate this interaction are yet to be defined. In vertebrates, Nup153 is a mobile nucleoporin that is located in the inner ring of the NPCs and it associates with the Ig fold of both type A and B lamins (Al-Haboubi et al., 2011). Thus, Nup153 is believed to be involved in the interaction between the lamina and NPCs (Smythe et al., 2000; Walther et al., 2001), although other proteins may also participate in this interaction. Plants have a functional homolog of Nup153, Nup136 that was proposed to link the NPCs to the plant lamina (Tamura and Hara-Nishimura, 2011, 2013). Nup136 is unique to higher plants and while it shares no sequence similarity with vertebrate Nup153, it has some characteristics in common with the latter and it is also mobile, as demonstrated by FRAP analysis (Tamura et al., 2010). Although a direct interaction of NMCPs with Nup136 has not been proved, over-expression and down-regulation experiments showed that Nup136 and *linc/crwn* mutants have similar morphological alterations in the nucleus (Dittmer et al., 2007; Tamura et al., 2010; Sakamoto and Takagi, 2013), suggesting that both proteins interact and regulate nuclear morphology, and that Nup136 also links the NPC to the lamina (Tamura and Hara-Nishimura, 2011). It has been speculated that nuclear pore anchor (NUA), a nucleoporin of the nuclear pore basket, is involved in establishing nuclear architecture (Xu et al., 2007a, b). NUA is the plant homolog of the vertebrate nucleoporin Tpr, a long coiled coil protein that constitutes the scaffold of the nuclear pore basket (Strambio-De-Castillia et al., 2010). NUA accumulates in the inner side of the NE but its interaction with NMCP proteins and its role in nuclear organization have yet to be defined.

## THE LAMINA AND NUCLEOCYTOPLASMIC BRIDGING COMPLEXES IN PLANTS

Amongst the integral proteins of the INM that bind to lamins in the metazoan lamina are the SUN domain proteins, which interact with the KASH domain proteins in the perinuclear space of the ONM to form the LINC complexes. The latter constitute the core of the connection between the nuclear lamina and the perinuclear cytoskeleton, forming a nucleocytoplasmic continuum. The LINC complexes fulfill a mechanical role in nuclear positioning and movement, centrosome attachment to the ONM, linking the nucleoskeleton to the cytoskeleton, and telomere positioning during meiosis, as well as participating in non-mechanical events regulating nuclear shape and size, and acting as specialized NE receptors (Tzur et al., 2006; Razafsky and Hodzic, 2009; Starr and Fridolfsson, 2010; Rothballer and Kutay, 2013; Sosa et al., 2013; Tapley and Starr, 2013; Stewart and Burke, 2014).

SUN proteins are highly conserved in eukaryotes, and while single cell eukaryotes have one SUN protein, *C. elegans* and *Drosophila* have two, and mammals and plants have multiple SUN proteins expressed at different times during development. The two major mammalian SUN proteins, SUN1 and SUN2, are widely expressed, while SUN3, 4, and 5 expression is restricted to the testis (Starr and Fridolfsson, 2010; Sosa et al., 2013; Zhou and Meier, 2013). Metazoan SUN proteins have a conserved domain layout, with a nucleoplasmic N-terminus that interacts directly with the Ig fold of lamins, followed by a transmembrane domain, a predicted coiled coil segment that localizes to the perinuclear space and allows trimerization, and a C-terminal SUN domain of about 175 amino acids that interacts with the KASH domain of KASH proteins (Tzur et al., 2006; Sosa et al., 2012). KASH domain proteins are more diverse. Yeast and *Drosophila* have two KASH proteins, *C. elegans* has three and mammals have six such proteins, called nesprins (Zhou and Meier, 2013). KASH proteins have an N-terminal cytoplasmic segment of varying size, structure and function, and a conserved C-terminal KASH domain that includes a transmembrane domain and a luminal domain of 20–30 amino acids. A PPPX motif can be found at the end of the C-terminus of typical KASH proteins, with conserved hydrophobic residues that lie upstream of it, both features that are essential for interactions with KASH and SUN domains (Rothballer and Kutay, 2013). In the formation of metazoan LINC complexes, the SUN proteins in the INM form homotrimers through the association of their coiled coil domains. The three adjacent SUN domains form clover-like trimers that interact with the KASH domains of three independent proteins anchored to the ONM, and binding is further stabilized by the formation of an intermolecular disulphide bond that covalently links the SUN and KASH domains. The cytoplasmic domains of KASH proteins anchored in the ONM interact with microtubule motors or actin filaments at the nuclear surface in order to move nuclei or to generate forces at the NE (Starr and Fridolfsson, 2010; Sosa et al., 2012, 2013; Tapley and Starr, 2013).

Plants encode up to five different SUN domain proteins that can be categorized into two classes: the canonical C-terminal SUN proteins SUN1 and SUN2 that are the structural homologs of the animal and yeast SUN1 and SUN2 proteins, and that contain a conserved domain layout with a NLS in the N-terminal domain, a transmembrane domain, a coiled coil domain and a highly conserved C-terminal SUN domain (Graumann et al., 2010, 2013; Murphy et al., 2010; Oda and Fukuda, 2011); and the plant prevalent mid-SUN3 proteins that contain three transmembrane domains, one at the N- and two at the C- terminus, as well as a SUN domain in the middle of the protein, which is followed by a highly conserved PAD (PM3-associated) domain of unknown function and a coiled coil domain (Murphy et al., 2010; Graumann et al., 2013). Unlike C-terminal SUNs, mid-SUN proteins have not yet been physiologically investigated. AtSUN1 and AtSUN2 are highly immobile intrinsic components of the NE, as demonstrated by FRAP analysis (Graumann et al., 2010). Moreover, recent results indicate that they interact with NMCP proteins (Graumann, 2014), suggesting an association with the lamina. They also form homomers and heteromers *in vivo* through the interaction of their coiled coil domains, as demonstrated by FRET (Graumann et al., 2010), indicating that they may function as multimer complexes. The predicted 3D structure of the SUN domain of AtSUN1 revealed that the essential structures and amino acids involved in KASH binding are conserved in relation to HsSUN2, but not the residues dispensable for the SUN–KASH interaction (Zhou and Meier, 2013). AtSUN1 and AtSUN2 are involved in regulating nuclear shape (Oda and Fukuda, 2011; Zhou et al., 2012), anchoring protein complexes to the NE (Zhou et al., 2012), and linking the nucleus to cytoskeleton (Tamura et al., 2013). Hence, like their animal counterparts, plant SUN proteins appear to be key components involved in different protein networks, including the lamina, NE and nucleocytoplasmic bridging complexes.

Despite the conservation of SUN proteins, plants do not contain homologs of the opisthokont KASH proteins, although novel plant-specific SUN-interacting proteins were identified in *Arabidopsis*, WIPs (tryptophan-proline-proline [WPP] domain interacting proteins). WIPs are ONM anchored proteins with a cytoplasmic coiled coil domain, a transmembrane domain and a C-terminal tail in the perinuclear space, and they have a terminal conserved VPT motif that is essential for the interaction with SUN proteins (Zhou et al., 2012). *Arabidopsis* has three WIP homologs AtWIP1, AtWIP2, and AtWIP3 that interact with SUN proteins through the SUN domain. Accordingly, the SUN–WIP bridge would be the plant counterpart of the SUN–KASH bridge that forms the metazoan LINC complex. WIP proteins also redundantly anchor the RanGAP (Ran GTPase activating protein) to the NE through an interaction involving the N-terminal specific WPP domain of RanGAP and the coiled coil domain of WIPs. In this way, the SUN–WIP interaction provides a NE bridging complex and the anchoring of RanGAP to this structure suggests additional functions for these complexes (**Figure 4**; Zhou et al., 2012; Zhou and Meier, 2013). The possibility that the SUN–WIP bridges could connect with the cytoskeleton was recently enhanced with the discovery of a new type of plant nucleocytoplasmic linker involved in the regulation of nuclear shape and movement. This linker consists of a plant-specific myosin motor (Myosin Xl-i) that binds to both the actin filaments of the perinuclear cytoskeleton and the ONM WIT (WPP domain interacting tail anchored) proteins, with a similar domain organization to WIPs (Zhao et al., 2008), and which in turn interacts with the SUN–WIP bridge (**Figure 4**; Tamura et al., 2013).

Hence, NE bridging complexes connected to the lamina exist in plants. In this regard, while the INM components of these complexes are conserved in plants, the SUN proteins, their ONM partners are plant-specific and share no similarity with the KASH proteins. Thus, while the linkers of the nucleoskeleton to cytoskeleton (LINC) complexes are conserved in animals, they appear to have partially diverged in plants. The reported plant LINC complexes are involved in connecting the nucleus with actin filaments through a myosin motor that interacts with a plant-specific ONM protein, a mechanism that is unique to plants (Tamura et al., 2013). They are also implicated in the control of nuclear shape and movement in response to environmental stimuli, yet not in light-induced nuclear movement (Tamura et al., 2013). Hence, different mechanisms driving nuclear movement apparently exist in plants. As indicated above, plant LINC complexes also perform other unique functions, such as the anchoring of RanGAP to the ONM (Zhou et al., 2012).

## PERSPECTIVES

Significant advances have been made in recent years in terms of the structural characterization of the plant lamina (Fiserova et al., 2009), as well as in the identification of its major protein components, the NMCP proteins considered to be functional analogs of metazoan lamins (Ciska and Moreno Díaz de la Espina, 2013). Nevertheless, considerable work is still needed, not only to advance in the knowledge of the mechanisms of assembly of NMCP proteins in the lamina and their functions but also, to fully understand the complete protein composition of this structure, the interactions therein and the roles it fulfills. One of the issues pending is the determination of the set of plant-specific proteins that drive the association of the lamina with the INM, NPCs, nucleocytoplasmic bridging complexes and chromatin, and the molecular interactions responsible for the association of these structures with NMCPs. The characterization of these proteins would represent an important advance in our understanding of the composition of the plant lamina and the protein interactions therein.

The bridging complexes that connect the plant lamina with the cytoskeleton are now beginning to be characterized. Their INM components (SUN proteins) are conserved, while those associated with the ONM are plant-specific (WIP proteins), evidence that the eukaryotic LINC complexes have partially diverged (Zhou et al., 2012; Zhou and Meier, 2013). The plant SUN–WIP core complexes are involved in connecting the nucleoskeleton with the actin cytoskeleton through a mechanism other than that involving animal LINC complexes. This interaction involves a plant-specific myosin motor that interacts with both actin filaments and a WIT protein, the latter associating with the WIP core protein of the complex (Tamura et al., 2013). The SUN–WIP complexes are also involved in anchoring protein complexes to the NE, like RanGAP, which fulfills plant-specific functions (Zhou et al., 2012). Apart from the association of NMCPs and SUNs (Graumann, 2014) the mechanisms that are responsible for stabilizing these complexes in the lamina remain unknown. The γ-Tubulin complexes (γ-TuCs) that nucleate MTs at the ONM are speculated to associate through the interaction of the small protein components of these complexes, GIPs (GCP3-interacting proteins). GIPs are required for correct γ-TuC localization at the NE, partnering TSA1 (TonSoKu [TSK]-associating protein 1), which has been proposed to interact with SUNs in the perinuclear space through its VIPT motif (**Figure 4**; Batzenschlager et al., 2013). Hence, the nucleocytoplasmic linker involved in the association of these complexes appears to display a quite diverse composition.

Despite the advances in our understanding of the plant lamina in the last few years, we still have very limited information about this NE component, and there are still many questions to be answered regarding the composition and functions of this structure. Which proteins link the NMCP-based lamina to the INM, nucleocytoplasmic linkers and chromatin? How is the plant lamina involved in chromatin tethering, organization and regulation? What are the functional capacities of the plant lamina? The study of the plant lamina is a field with great potential in plant nuclear biology, which will shed light on the mechanisms regulating nuclear shape and architecture, the connection of the nucleoskeleton to the cytoskeleton, nuclear positioning and movement, chromosome organization and positioning, gene expression, etc.

## Conflict of Interest Statement

The authors declare that the research was conducted in the absence of any commercial or financial relationships that could be construed as a potential conflict of interest.
